# Exploring factors associated with research involvement of undergraduate students at the College of Medicine and Health Sciences, University of Rwanda

**DOI:** 10.1186/s12909-021-02662-3

**Published:** 2021-04-26

**Authors:** Eric Mugabo, Lotta Velin, Richard Nduwayezu

**Affiliations:** 1 University of Rwanda, College of Medicine and Health Sciences, Kigali, Rwanda; 2 WHO Collaborating Centre for Surgery and Public Health, Skane University Hospital, Department of Clinical Sciences, Lund University, Lund, Sweden

**Keywords:** Research, Undergraduate, Rwanda, Medical student, Barriers, Facilitators

## Abstract

**Background:**

Early involvement of students in research processes is an important step in professional development and can increase the academic output of the university. Previous studies indicate low research involvement amongst undergraduate students, however limited research has been done in sub-Saharan Africa. This study aimed to describe the level of research involvement amongst undergraduate students at the College of Medicine and Health Sciences (CMHS) at University of Rwanda (UR) and to assess factors associated with research involvement.

**Methods:**

This cross-sectional study covered the three CMHS campuses. A survey was shared in class WhatsApp groups from July to September 2020. Data were analyzed using Stata IC 16.0 with descriptive statistics and Fisher’s exact test. *P*-values < 0.05 were considered statistically significant.

**Results:**

In total, 324 students participated with the mean age being 23.3 (standard deviation 2.27). Males constituted 65.1% of respondents vs. 33.3% females. The largest portion of respondents were from the School of Medicine and Pharmacy (46.6%), and Medicine was the most frequent department (33.3%). On a Likert scale from 1 to 10, 60.0% of the respondents thought that research was 10/10 important for undergraduate students, with the mean value being 8.8. Rating their interest in taking part in research during undergraduate studies, 48.2% scored it 10/10, with the mean value being 8.57. 80.3% of respondents had attended a research module, course, or workshop; however, only 48.8% had participated in a research project and 72.0% of them had been involved in data collection. Inadequate knowledge about research processes and lack of mentors were the main barriers to research participation in 48.0 and 40.2% of respondents respectively. Establishment of a UR-Undergraduate research support center (77.2%), and involving students in ongoing UR projects (69.4%) were the most frequent suggestions to improve students’ research participation.

**Conclusion:**

Undergraduate students at the CMHS in the UR have a large research interest, yet their involvement is currently low. Limited knowledge about research processes and shortage of mentors remains potent barriers to participation. Inviting undergraduate students to partake in ongoing projects and establishing a UR undergraduate research support center are recommended to strengthen undergraduate research experience at the UR-CMHS.

**Supplementary Information:**

The online version contains supplementary material available at 10.1186/s12909-021-02662-3.

## Introduction

Low and middle-income countries (LMICs) are still facing a large burden of diseases and high mortality rates; yet the majority of global health research stems from high-income countries [1, 2]. This is associated with inadequate health care systems and suboptimal quality of healthcare services in LMICs; indicating a need for contextual research to guide improvements in healthcare delivery to close the gap between research and health outcomes [3].

Research involvement can help develop critical thinking skills and early participation of students in research has been demonstrated as an important step for spurring further research interest and help them further on the path towards an academic career [4–7]. Moreover, engaging students in research can be mutually beneficial, providing teaching opportunities for mentors, the possibility of increased academic output for the university in question, and creates local evidence that can be used to guide health policy [7–9]. However, students in medicine and health sciences in LMICs face several barriers to research involvement including the lack of knowledge and lack of time, as well as institutional barriers such as lack of mentoring, limited database access, and lack of funding [10–15]. Undergraduate students’ research involvement varies across universities and is associated with several factors including male gender, completing degrees in specific disciplines, and having previously completed a curricular research project [16].

Rwanda is a low-income country located in East Africa, and the University of Rwanda (UR) is its largest public academic institution. The UR envisions being an academically excellent research-led institution that is locally relevant and internationally recognized. It further realizes that it is essential to create an enabling environment for research [17]. Undergraduate students at the UR College of Medicine and Health Sciences (CMHS) have a research introductory course that covers writing a research proposal, study design, data collection, and data analysis. After completing this module students seldom get opportunities to practice what they have learned, except for at the end of their undergraduate studies when they have to write a thesis as partial fulfillment of their academic requirement [18].

In 2019, a study by Habineza et al. assessed attitudes of the perceived importance and barriers to research amongst Rwandan medical graduates (interns) and pediatric residents, however, this study did not cover all CMHS departments and focused only on medical graduates [19]. Hence, our study aimed to provide a deeper understanding of the factors associated with research involvement among CMHS undergraduate students at UR and assess their involvement in research projects. The results of this study will be used to propose recommendations that will further improve the research experience among CMHS undergraduates.

## Methods

### Study design and location

UR is structured in six different colleges, where the CMHS is one [20]. The CMHS has five schools: Medicine and Pharmacy, Dentistry, Nursing & Midwifery, Health Sciences, and the School of Public Health. CMHS head office is located in Remera, in the capital city of Kigali. This cross-sectional study took place in the three UR Campuses: the Remera campus in Kigali, the Rwamagana campus, located at approximately 2 h driving distance from Kigali, and in the campus of Huye one of the six secondary cities in Rwanda. Data from the UR registrar office shows that 3559 students were enrolled at CMHS in the academic year 2019–2020.

Bachelor programs at CMHS-UR are covered in a period of 4 years except medicine and pharmacy which take 5 years. The CMHS also offers three-year long academic programs at an undergraduate level which lead to “advanced diplomas”. An introductory module about biomedical research is in place for all CMHS undergraduate students, and is mostly covered in the second year, although some programs have it in year one.

### Subjects and sampling process

Using a sample size calculator with confidence level 95%, margin error 5%, and population size 3559, the sample size was calculated to be 347. Sample participants were obtained by convenience sampling where students who were willing to participate filled the survey form.

Inclusion criteria to participate in the study were: being registered in any undergraduate program in CMHS during the 2019–2020 academic year; having completed at least 1 year of courses at CMHS; being above 18 years of age; and having accepted to sign the consent form. Exclusion criteria were: students who were registered for the academic year 2019–2020 but had suspended their studies; year 1 students; and students aged 18 or younger at the time of the study.

### Survey tool

This study was done through an online Google form (Supplement 1). Survey questions were developed based on the findings of a preliminary literature review on research involvement amongst undergraduate students in medicine and health sciences [16, 19, 21, 22]. The form was piloted by 10 non-CMHS students to assess its validity, and feedback received was used to improve the form. The form consisted of five sections: introduction/demographics, attitudes towards research, the extent of research involvement, factors associated with research involvement, suggestions of what can be done to improve research involvement. There were in total 19 questions, of which five were multiple choices, seven checkboxes, four Yes/No questions, and two questions with 1–10 graded Likert scales and one short answer question.

### Ethical considerations

Before starting data collection process, this study received ethical approval from the University of Rwanda, College of Medicine and Health Sciences Institutional Review Board (CMHS-IRB), (No 082/CMHS IRB/2020) and all methods were performed in accordance with CMHS-IRB guidelines and regulations. Taking part was voluntary, informed consent was obtained from all participants; respondents under the age of 18 were not eligible to participate. The form collected no personal identifications and respondents could stop filling the form whenever they wanted to do so. Data were stored in a password-protected Google Drive that could be only accessed by the research team.

### Data collection and validation

Data was collected for 3 months (July, August, and September 2020) through an online Google form that was shared with all eligible students in their respective classes’ WhatsApp groups. WhatsApp was preferred over emails due to the fact it is the commonly accessed social media tool for daily class updates and usually, students could not check their emails frequently. The form settings did not allow respondents to submit an incomplete form and each respondent was allowed to fill the form only once to avoid duplication of answers.

### Statistical analysis

Collected data were cleaned; answers, where respondents were able to choose more than one option, were split and coded as “Yes” or “No”. Data were analyzed using Stata IC 16.0 with descriptive statistics (mean/median and standard deviation/interquartile range,) and Fisher’s exact test to assess which factors were associated with research interest and experience. *P*-values < 0.05 were considered statistically significant. Students in the medical degree program were compared with students in other programs, grouped as “non-medical students”.

## Results

### Descriptive statistics and baseline population comparisons

A total of 324 students participated in this study, equivalent to 9.1% response rate. The majority of respondents were males, (*n* = 211, 65.1%), (*n* = 108, 33.3%) were females and (*n* = 5, 1.5%) preferred not to disclose their gender (Table 1). The mean age was 23.3 (standard deviation 2.27). Most respondents were from the School of Medicine and Pharmacy (*n* = 151, 46.6%), followed by Nursing and Midwifery (*n* = 91, 28.1%), and Health Sciences (*n* = 33, 10.2%). The most frequent department was Medicine (*n* = 108, 33.3%), followed by General Nursing (*n* = 62, 19.1%), and Pharmacy (*n* = 37, 11.4%). Nearly half of the respondents were in the third year of study (*n* = 153, 47.2%), and most were students in the fourth (*n* = 92, 28.4%) or second year (*n* = 59, 18.2%) of study.

**Table 1:** Demographic characteristics of survey respondents

Demographic characteristic	Total ***n*** = 324
Gender (n, %)	
- Female	108 (33.3)
- Male	211 (65.1)
Age (mean, standard deviation)	23.3 (2.27)
School (n, %)	
- Medicine and Pharmacy	151 (46.6)
- Nursing and Midwifery	91 (28.1)
- Health Sciences	33 (10.2)
- Public Health	31 (9.6)
- School of Dentistry	18 (5.6)
Academic year (n, %)	
- Second	59 (18.2)
- Third	153 (47.2)
- Fourth	59 (18.2)
- Fifth	20 (6.2)

### Attitudes and perception towards research

On a Likert scale ranging from 1 to 10, the majority (*n* = 194, 60.0%) of respondents thought that research was 10/10 important for undergraduate students, with the mean value being 8.8 (standard deviation 1.97; 8.8 ± 1.93 for males vs 8.90 ± 1.88 for females). Nearly half of the respondents (*n* = 156, 48.2%) ranked their interest in taking part in research during undergraduate studies as 10/10, with the mean value being 8.57 (standard deviation 1.94, 8.5 ± 1.91 for males vs 8.6 ± 2.00 for females).

### The extent of research involvement

Regarding research involvement, 80.3% (*n* = 260) of respondents had attended a research module, course, or workshop; however, only 48.8% (*n* = 158) had participated in a research project. Comparing research engagement among academic levels, (*n* = 81, 72.3%) of senior students (academic year 4 or 5) had engaged in research vs (*n* = 77, 36.3%) of junior students (academic year 3 and 2), *p* = 0.000. More than half of the students who had been involved in research had worked on research projects through UR (*n* = 84, 51.5%), 38.0% (*n* = 62) had engaged in research through a student-led organization, and 25.2% (*n* = 41) through another academic institution.

For students who had previously participated in research, most (*n* = 103, 72.0%) had been involved in data collection, followed by involvement in study design and writing a study protocol (*n* = 60, 42.0%). Few students had been able to take part in the dissemination of data, with 11.2% (*n* = 16) having presented at a conference and 10.5% (*n* = 15) having been a co-author on a publication in a peer-reviewed journal.

When comparing medical students and non-medical students, 44.9% (*n* = 97) of non-medical respondents had participated in research vs. 41.7% (*n* = 45) of medical respondents, *p* = 0.635 (Table 2). Most students who had published in a peer-reviewed journal were non-medical students (*n* = 15, 6.9%) vs (*n* = 5, 4.6%) of medical students, although this difference was non-significant *p* = 0.469.

**Table 2:** Comparative analysis of research interest and involvement amongst medical students and non-medical students

	Total	Medical students	Non-medical students	*p*-value
Attitudes towards research				
Importance of research (mean score out of 10)	8.77	8.66	8.89	0.140
Interest in research (mean score out of 10)	8.57	8.59	8.56	0.058
The extent of research involvement				
Have attended a research module (n, %)	259 (81.2)	87 (80.6)	172 (81.5)	0.880
Have participated in a research project (n, %)	158 (49.5)	50 (46.3)	108 (51.2)	0.478
Have participated in … (n, %)^a^	103 (72.0)	34 (73.9)	69 (71.1)	0.843
Data collection	60 (42.0)	20 (43.5)	40 (41.2)	0.857
Study design and writing a study protocol	49 (34.2)	18 (39.1)	31 (32.0)	0.452
Writing an abstract	48 (33.6)	21 (45.7)	27 (27.8)	0.039*
Data management and analysis	32 (22.4)	10 (21.7)	22 (22.7)	1.000
Applying for ethical approval	31 (21.7)	10 (21.7)	21 (21.7)	1.000
Grant writing	22 (15.4)	7 (15.2)	15 (15.5)	1.000
Writing of a manuscript				
Presenting at a conference	16 (11.2)	5 (10.9)	11 (11.3)	1.000
Being a co-author on a publication	15 (10.5)	5 (10.9)	10 (10.3)	1.000

*p-value < 0.05

^a^For students who had previously participated in research

More than half of males, (*n* = 111, 52.6%) had participated in a research project vs 43.5% (*n* = 47) of females, *p* = 0.023 (Table 3). More females (*n* = 7, 6.5%) had published in a peer-reviewed journal than males (*n* = 13, 6.2%), although these differences were not statistically significant (*p* = 0.478 and *p* = 1.00). Only 5.4% (*n* = 6) of senior students vs 6.6% (*n* = 14) of junior students, had published in a peer-reviewed journal, *p* = 0.810.

**Table 3:** Comparative analysis of research interest and involvement amongst males and females

	Total	Females	Males	*p*-value
Attitudes towards research				
Importance of research (mean score out of 10)	8.8	8.9	8.8	0.098
Interest in research (mean score out of 10)	8.57	8.5	8.6	0.703
The extent of research involvement				
Have attended a research module (n, %)	260 (80.3)	83 (76.8)	176 (83.4)	0.174
Have participated in a research project (n, %)	158 (48.8)	47 (43.5)	111 (52.6)	0.023*
Have participated in … (n, %)^a^	103 (72.0)	35 (77.8)	68 (69.4)	0.324
Data collection	60 (42.0)	15 (33.3)	45 (45.9)	0.202
Study design and writing a study protocol	49 (34.3)	16 (35.5)	33 (33.7)	0.851
Writing an abstract	48 (33.6)	19 (42.2)	29 (29.6)	0.182
Data management and analysis	32 (22.4)	7 (15.6)	25 (25.5)	0.203
Applying for ethical approval	32 (21.7)	6 (13.3)	25 (25.5)	0.127
Grant writing	22 (15.4)	4 (8.9)	18 (18.4)	0.212
Writing of a manuscript	16 (11.2)	4 (8.9)	12 (12.2)	0.776
Presenting at a conference	15 (10.5)	3 (6.7)	12 (12.2)	0.390
Being a co-author on a publication				

*p-value < 0.05

^a^For students who had previously participated in research

### Skills and benefits acquired through research involvement

For students who had participated in research, 77.9% (*n* = 113) reported that they were able to understand the research process, and 56.6% (*n* = 82) reported that they understood how scientists work on problems. Other skills and benefits reported to be acquired through involvement in research were getting networking opportunities (*n* = 53, 36.6%), the ability to present research in a conference (*n* = 39, 26.7%), getting publication opportunities (*n* = 24, 16.6%) and the ability to write a research manuscript (*n* = 22, 15.2%).

### Barriers towards participation in research

**Fig. 1:**
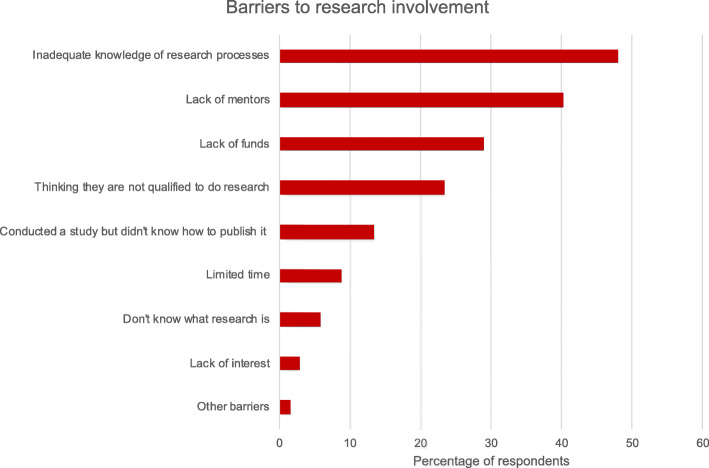
Barriers to research involvement, with the reported frequency amongst survey respondents

The most important barrier (Fig. 1) towards participation in research was students feeling that they had inadequate knowledge about research processes (*n* = 154, 48.0%). Other important barriers were lack of mentors (*n* = 129, 40.2%), lack of funds (*n* = 93, 29.0%), and undergraduate students thinking that they are not qualified to do research (*n* = 75, 23.4%).

### Suggestions of what can be done to improve research involvement

**Fig. 2:**
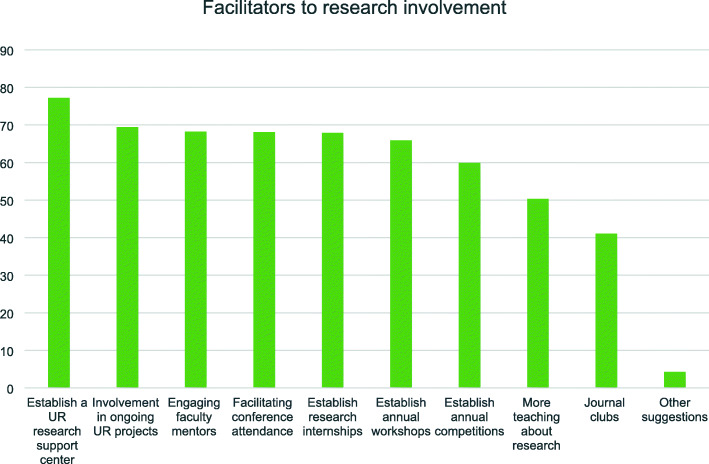
Facilitators to research involvement, with the reported frequency amongst survey respondents

When students were asked to suggest what can be done to improve their research involvement (Fig. 2), the most common suggestion received was to establish a UR-Undergraduate research support center (*n* = 250, 77.2%), followed by involvement of students in ongoing UR research projects (*n* = 225, 69.4%), and encouraging faculty members to mentor students (*n* = 221, 68.2%). Fourteen respondents made other suggestions including increasing funding for research projects by availing research grants for undergraduate students.

## Discussion

In this cross-sectional study, we found that research interest among undergraduate students in medical and health sciences in Rwanda is widespread, but research experience is still limited. More than half of the respondents (*n* = 194, 60.0%) believed that it is important to engage in research during undergraduate studies, and 48.2% (*n* = 156) expressed a large research interest. Most students had attended a research module or workshop (*n* = 260, 80.3%), but only 48.8% (*n* = 158) had taken part in a research project, most commonly through UR. The discrepancy between research interest and research experience was even more pronounced amongst female respondents, where 43.5% had taken part in a research project, compared to 52.6% of males, despite equivalent levels of research interest (*p* = 0.023). Among students who had taken part in research projects, most (*n* = 103, 72.0%) had been involved in data collection, followed by abstract writing (*n* = 49, 34.3%). Only 6.2% (*n* = 20) of students had been part of a publication in a peer-reviewed journal, of which 40.0% (*n* = 8) had more than one publication, with three respondents having more than four publications.

This study found that CMHS-UR undergraduate males and females have equivalent interests in research and perceive the importance of research at similar levels, however, more male students had attended a research course/workshop (83.4% vs 76.8%, *p* = 0.174) or had been involved in research project (52.6% vs 43.5%, *p* = 0.0203) projects than female students. Similar results have been reported in previous literature, where women are underrepresented in research both at the undergraduate level as well as the post-graduate level in sub-Saharan Africa and beyond [16, 23, 24]. This disparity is likely multifactorial, however may be overcome if targeted efforts are made, with previous studies indicating increased research participation and academic progress through gender-specific mentorship programs [25]. With women’s career progression still described as a “leaky pipeline” [26, 27], and the number of women reaching high leadership positions still low, it is important to understand if and how gender disparities at the most junior research level can be addressed.

Multiple barriers exist for CMHS undergraduate students interested in engaging in research. Limited understanding of the research process was found to be the most potent (*n* = 154, 48.0%). Despite most (*n* = 260, 80.3%) having attended a research course or workshop, students who had been involved in research claimed to have understood research processes (*n* = 113, 77.9%) and how scientists work on problems (*n* = 82, 56.6%), which is similar to the Rwandan study by Habineza et al., that showed that learning how to conduct research was the primary perceived benefit of students’ research involvement [19]. This is similar to findings in a previous cross-sectional study conducted with 687 clinical students in Pakistan, where lack of knowledge was the most common barrier for students to do research [10], and studies in other LMICs where students reported limited research skills [12, 14]. Interestingly, 23.4% (*n* = 75) of the respondents in our study said that they think that they are not qualified to do research at the undergraduate level. This could also be a symptom of limited knowledge about research processes which may lead to uncertainty regarding who is qualified and who is not.

Other barriers, such as lack of mentors and lack of funds, identified in this study are indicative of limited institutional research infrastructure, which mirror previous findings in similar studies in other LMICs [11–14]. Students may be unable to do research projects on their own but if they are in a supportive learning environment where research opportunities are available, the threshold to engage in research could be lowered [14]. To capitalize on students’ interest in research, it is also important to ensure that students have access to research opportunities at all parts of the process, where our findings, as well as previous studies in LMICs, indicate that opportunities are often limited to data collection, and only a minor proportion get the chance to be a part of a peer-reviewed publication [19, 28]. Currently, University of Rwanda has a research directorate that facilitates research, yet undergraduate involvement is still limited [17]. Therefore, we recommend the formalization of such a platform at the UR through the establishment of a UR-Undergraduate research support center, as supported by the majority of the survey respondents. Such a center exists for example at the University of California Davis campus and encourages and facilitates research opportunities, offering awards and activities to support undergraduate research across the university [29]. Additionally, we recommend utilizing students’ interest and involving students in ongoing UR research projects and encouraging faculty members to mentor students to strengthen UR undergraduate’s involvement in research. With previous studies indicating that senior faculty may not feel confident to act as mentors, increasing resources and support for mentors may also be of importance [30]. However, students’ research involvement should not be restricted to single tasks, such as data collection, but rather an invitation to join the research team and take part in the full process – so that students can begin their path in growing to become independent researchers.

With shortage of available mentors, models to strengthen undergraduate research involvement and increase dissemination of students’ research can also pioneered by fellow students. One such example is the undergraduate research committee established at the Alfaisal University, Riyadh, Saudi Arabia, through which more than 60% of graduate students got involved a research program, and 50% published in a peer reviewed journal [31]. Similarly, “advisory peer review boards” where students provide constructive feedback on formatting, language, and offer suggestions to increase the chances of the manuscript being peer-reviewed and accepted by an indexed journal offer a potentially scalable model to increase access to research mentorship [32].

### Study limitations

Throughout this study, the lockdown due to COVID-19 and the corresponding closure of the UR provided considerable challenges, that were not expected during the preparation of the study. We started data collection in July when the country was in partial lockdown, and students were no longer on the campus and no online academic activities were taking place. In addition, students were located in different geographical locations with limited internet coverage. In some cases, financial constraints hindered students from taking part. For example, some students expressed that taking part was not free, since internet bundles needed to fill in an online form cost money. These factors may explain the fact that we did not reach the intended sample size.

This study was limited to cross-sectional observation, and respondents were not able to express their opinions in free text. To provide a more in-depth understanding of barriers and solutions to undergraduate participation in research, a complementary qualitative study could be of interest. It frequently happens that survey respondents give positive answers (acquiescence bias), to limit participants from giving what they think research team wants all responses were anonymized.

Another limitation could be that students may not be inclined to open voluntary links, where no incentives are provided; and those who do take part may have a specific interest in research. Finally, UR is the largest academic institution in Rwanda with more comprehensive research infrastructure than most other institutions for medicine and health sciences, so it is likely that the results could differ in other parts of the country.

## Conclusion

This study analyzed factors associated with research involvement among CMHS undergraduate students at UR. Most students at the CMHS UR had taken part in a research module, course, or workshop, yet students felt that they lack knowledge of the research process, and some students do not think that they are qualified to carry out research. To meet the large interest in research at the undergraduate level, efforts should be made to make research opportunities more accessible, potentially through the establishment of a UR-Undergraduate Research Support Center.

## Supplementary Information

**Additional file 1.** Questionnaire.

## Data Availability

Data collected during this study is safely protected in Google drive. The datasets used and/or analysed during the current study are available from the corresponding author on reasonable request.

## Abbreviations

CMHS: College of Medicine and health Sciences
IRB: Institutional Review Board
LMICs: Low and Middle Income Countries
UR: University of Rwanda
