# Supplementary material for: Exploring factors associated with research involvement of undergraduate students at the College of Medicine and Health Sciences, University of Rwanda
Source: BMC Med Educ. 2021 Apr 26;21:239. doi: 10.1186/s12909-021-02662-3 (PMC8072743; doi:10.1186/s12909-021-02662-3)
Supplement: Supplementary file 1 — Additional file 1. Questionnaire. [file 12909_2021_2662_MOESM1_ESM.docx]

## **EXPLORING FACTORS ASSOCIATED WITH RESEARCH INVOLVEMENT AMONG UNDERGRADUATE STUDENTS AT THE COLLEGE OF MEDICINE AND HEALTH SCIENCES, UNIVERSITY OF RWANDA**

## **QUESTIONNAIRE**

1. ENGLISH QUESTIONNAIRE

**Introduction**

1. Age (number only):
2. Sex:

- Male
- Female

1. School:

- Medicine and Pharmacy
- Dentistry
- Nursing and Midwifery
- Health sciences
- School of public health

1. Department:

- Medicine
- Pharmacy
- Clinical psychology
- Dental therapy
- Dental surgery
- Nursing
- Midwifery
- Mental health nursing
- Environmental health
- Nutrition and dietetics
- Laboratory sciences
- Medical imaging
- Ophthalmology
- Occupation therapy
- Anesthesia
- Orthopedic technology
- Physiotherapy

1. Academic level / year of study

2 3 4 5

**Attitudes and perception towards research**

1. How much do you think that research is important for undergraduate students? (1 = not at all important, 10 = very important)
2. To what extent are you interested in taking part in research during your undergraduate studies? (0= not interested, 10 = very interested)
3. If you identify a problem, how do you investigate it?
4. Read about it
5. Ask friends
6. Carry out a research study about it
7. Nothing
8. Other (please specify)

**Extent of research involvement:**

1. Have you attended any research course/module or workshop?
2. Yes
3. No
4. Have you ever engaged or participated in any research projects through a given institution?
5. Yes
6. No
7. If yes on question 10, what was the institution? (tick all that applies)
8. University of Rwanda
9. Other academic institution
10. Students led organization
11. Other ( Write yourself )
12. Have you ever carried out a research study or been a part of a research team?
13. Yes
14. No
15. If yes on question 12, tick all the processes that you were a part of
16. Grant writing
17. Study design and writing a study protocol
18. Applying for ethical approval
19. Data collection
20. Data management and analysis
21. Writing of an abstract
22. Writing of a manuscript
23. Disseminating results by presenting at a conference
24. Disseminating results by being a part of a publication
25. If yes to question 12, please tick all the benefits that applies to you after the research study
26. Understanding the research process
27. Understanding how scientists work on problems
28. Learning lab techniques
29. The ability to design a study and write a study protocol
30. The ability to apply for ethical approval
31. The ability to collect data
32. Developing skills in the interpretation of results
33. The ability to analyse data (quantitative or qualitative)
34. The ability to integrate theory and practice
35. The ability to write a research abstract
36. The ability to write a research manuscript
37. The ability to present research in a conference
38. Networking opportunities
39. Getting publication opportunities
40. Have you ever published any study?
41. Yes
42. No
43. If yes on question 15, how many publications do you have?
44. 1
45. 2
46. 3
47. 4
48. 5
49. More
50. If Yes on question 15, who were the collaborators of your research?
51. Classmates
52. University mentor / corresponding author
53. A friend
54. Research group
55. Others (specify)

**Factors associated with research involvement among UR CMHS undergraduates**

1. Which of the following barriers have you encountered when trying to do research? (tick all that apply)
2. I don’t know what research is
3. I had a research idea but I don’t have a mentor
4. I have not got enough knowledge about research processes (ethical approval, study design, data analysis, etc)
5. I don’t think I am qualified to do a research study (due to my academic degree)
6. I proposed a study, but I lacked the funds to conduct it
7. I conducted a study, but I didn’t know how to get it published
8. I am not interested in research
9. I do not have time to get involved in research
10. Other, please specify

**What can be done to improve research involvement for UR CMHS undergraduates?**

1. Suggestions of what can be done to improve research involvement among UR CMHS undergraduates. (tick all that apply)
2. Establishing UR undergraduate research support center
3. Encourage high academic profiled lecturers to mentor UR undergraduates in research
4. Organize annual research workshop for undergraduate students
5. Establish annual undergraduate research competitions
6. Involve UR CMHS undergraduates in ongoing research studies
7. Establish research internships for undergraduate students
8. Offer more teachings about research in undergraduate program
9. Facilitate undergraduate students to attend research conferences
10. Establish journal clubs (regular meeting to discuss about different studies to gain more understanding of research)
11. Others, Please specify
